# Methyl Gallate Attenuates Doxorubicin-Induced Cardiotoxicity in Rats by Suppressing Oxidative Stress

**DOI:** 10.1155/2021/6694340

**Published:** 2021-01-13

**Authors:** Akheruz Zaman Ahmed, Shakta Mani Satyam, Prakashchandra Shetty, Melanie Rose D'Souza

**Affiliations:** ^1^Department of Anatomy, Melaka Manipal Medical College (Manipal Campus), Manipal Academy of Higher Education, Manipal-576104, Karnataka, India; ^2^Department of Pharmacology, Melaka Manipal Medical College (Manipal Campus), Manipal Academy of Higher Education, Manipal-576104, Karnataka, India

## Abstract

Doxorubicin-induced cardiotoxicity is the leading cause of morbidity and mortality among cancer survivors. The present study was aimed to investigate the cardioprotective potential of methyl gallate; an active polyphenolic nutraceutical, against doxorubicin-induced cardiotoxicity in Wistar rats. Twenty-four female Wistar rats (150–200 g) were divided into four groups (*n* = 6) which consist of normal control (group I), doxorubicin control (group II), test-A (group III), and test-B (group IV). Group III and group IV animals were prophylactically treated with methyl gallate 150 mg/kg/day and 300 mg/kg/day orally, respectively, for seven days. Doxorubicin (25 mg/kg; single dose) was administered through an intraperitoneal route to group II, III, and IV animals on the seventh day to induce acute cardiotoxicity. On the 8^th^ day, besides ECG analysis, serum CK, CK-MB, LDH, AST, MDA, and GSH were assayed. Following gross examination of isolated hearts, histopathological evaluation was performed by light microscopy. A significant (*p* < 0.05) cardiac injury, as well as oxidative stress, was observed in doxorubicin control rats in comparison to normal control rats. Methyl gallate at both the doses significantly (*p* < 0.05) reduced doxorubicin-induced ECG changes, dyslipidaemia, and elevation of CK, CK-MB, LDH, AST, MDA and increased GSH level. Methyl gallate reversed the doxorubicin-induced histopathological changes in the heart. The present study revealed that methyl gallate exerts cardioprotection against doxorubicin-induced cardiotoxicity in female Wistar rats by suppressing oxidative stress. Our study opens the perspective to clinical studies for consideration of methyl gallate as a potential chemoprotectant nutraceutical in the combination chemotherapy with doxorubicin to limit its cardiotoxicity.

## 1. Introduction

Cancer survival rates are significantly improving mainly due to the reflection over the past several decades of improved screening, diagnostic imaging, and improvement of therapeutic modalities in oncology. However, this improved cancer survival rate is also associated with treatment-related toxicities that may significantly affect the patient's health and quality of life [1].

Doxorubicin (DOX), an anthracycline anticancer antibiotic, is a backbone in the chemotherapy regimens for different cancers including breast cancer and lymphoma [1–4]. Cardiotoxicity is the prime cause of morbidity and mortality due to DOX treatment among cancer survivors. [2, 5–7]. DOX-induced cardiotoxicity limits its therapeutic application to some extent [8]. Dose-dependent cardiotoxicity caused by DOX may occur early at the onset of treatment and even up to many years after completion of treatment [9]. The incidence of DOX-induced cardiotoxicity is highly variable amongst studies due to the different definitions of cardiotoxicity and the wide variety of pathology caused by DOX [1, 10–12].

Early detection of cardiotoxicity related to treatment and ongoing monitoring are key to avoiding unnecessary interruption of essential cancer therapy and in preventing long-term cardiotoxicity in cancer survivors [13]. In both clinical practice and basic cardiovascular research, electrocardiography (ECG) is widely used for monitoring cardiac dysfunction because ECG is affordable and commonly available. The electrocardiographic changes associated with cardiomyopathy caused by doxorubicin initially include multiple reversible arrhythmias, most commonly sinus tachycardia [14, 15]. Some of the electrocardiographic features observed later, with prolonged cardiotoxicity of doxorubicin, are associated with T-wave flattening, QT-interval prolongation, and also *R*-wave voltage loss [15, 16].

Although the investigation and research methodology have improved considerably over the years, the specific mechanism underlying DOX-induced cardiotoxicity remains elusive. DOX has great affinity towards negatively charged phospholipid cardiolipin, which is abundant in the inner mitochondrial membrane and accumulates it within the mitochondria of cardiomyocytes [17]. Many studies suggest that oxidative stress is the key mechanism for DOX-induced cardiotoxicity [18–20]. DOX causes excessive formation of reactive oxygen species (ROS) in the mitochondria causing oxidative damage to biological macromolecules, including lipids, proteins, and DNA, and affects the structure and functions of cardiac cell membranes [21]. Singal et al. reported that DOX reduces endogenous antioxidants and undergoes increase in lipid peroxidation that alters the cardiac function and with other toxicities [22]. Antioxidants are molecules that can suppress ROS and reduce oxidative stress damage. Even though extensive research works have been conducted to find effective treatment for cardiotoxicity caused by DOX, no such effective preventive treatment has yet been discovered.

Nutraceuticals are biologically active phytochemicals that possess health benefits. Currently, there is an increased global interest on the role of nutraceuticals in various diseases. Nutraceuticals field can be envisioned as one of the missing blocks in the health benefit of an individual. Methyl gallate (MG), an active polyphenolic compound, is methyl ester of gallic acid. The occurrence of MG has been reported in many plants including grape seeds, *Mangifera indica*, *Rosa rugosa*, *Schinus terebinthifolius*, and *Galla rhois* [23–25]. MG has been reported for significant biological activities including anticancer, antimicrobial, and anti-inflammatory properties and also has the great tendency to act as a cell adhesion inhibitor, cancer metastasis, skin papilloma promotion, and carcinomas [24, 26–28]. One of the studies reported that MG is a good free-radical scavenger that obstructs lipid peroxidation and has a defensive role from oxidative stress-induced DNA damage [29]. Khurana et al. reported that MG suppresses formation of intracellular ROS and enhances endogenous reduced glutathione in an animal model [30]. One of the studies reported that MG protects the cells from oxidative stress by downregulating intracellular ROS and upregulating HO-1, Nrf2, and PRDX3 [31]. Oidovsambu et al. reported that MG decreases ROS production and augment concentrations of total glutathione in hepatoma HepG2 cells in response to oxidative stress [32]. The role of MG in DOX-induced cardiotoxicity has not been reported yet. Hence, the aim of the present study was to investigate the cardioprotective potential of MG against DOX-induced cardiotoxicity in female Wistar rats.

## 2. Materials and Methods

### 2.1. Animals

Twenty-four adult female Wistar rats (age: 8–10 weeks and body weight: 150–200 g) bred in the Central Animal House, Manipal Academy of Higher Education (MAHE), Manipal, were housed in separate polypropylene cages. Animals were kept at temperatures (22–24°C), 12-hour light/12-hour dark cycle, and 40%–60% relative air humidity under standard conditions. Rats had continuous access to tap water with regular rat pellet diet on normal calories (Hindustan Lever Ltd., Mumbai, India). After randomization into different experimental groups, the rats were acclimatized to the laboratory conditions for one week before beginning the experiment. The experimental protocol was approved by the Institutional Animal Ethics Committee (IAEC/KMC/113/2019), and experiments were conducted in accordance with the ethical standards approved by the Ministry of Social Justice and Empowerment (Government of India) and the guidelines of Committee for the Purpose of Control and Supervision on Experiments on Animals (CPCSEA).

### 2.2. Chemicals

Active pharmaceutical ingredient form of doxorubicin and methyl gallate was obtained from Cipla Ltd., Goa (India), and Sigma-Aldrich-Merck Ltd., Bangalore (India), respectively. Assay kits for lipid profile, creatine kinase-MB (CK-MB), creatine kinase (CK), lactate dehydrogenase (LDH), and aminotransferase (AST) estimation were obtained from ASPEN Laboratories Ltd., New Delhi (India). Thiobarbituric acid (TBA), trichloroacetic acid (TCA), 5, 5′-dithiobis (2-nitrobenzoic acid) (DTNB), and reagents for histopathological analysis were procured from Sigma-Aldrich-Merck Ltd., Bangalore (India). All reagents were of analytical grade. Reagents were equilibrated at room temperature for 30 minutes before the biochemical estimations.

### 2.3. Rationale for Dose Selection of Doxorubicin

The widely used therapeutic dose of doxorubicin is 60–75 mg/m2 IV once every 21 days to treat varieties of cancers. This dose is equivalent to 20–25 mg/kg in rats [33].

### 2.4. Rationale for Dose Selection of Methyl Gallate

Two doses of MG (150 mg/kg & 300 mg/kg) were selected based on our results of the acute toxicity study (ATC method; OECD 423 guideline) for methyl gallate.

### 2.5. Experimental Design

We did cardiac screening for all the experimental animals using ECG to avoid inclusion of any animal having cardiac abnormality. Animals showing depressed ST segment/absence of P-wave/inverted P-wave/nonspecific ST segment/ST segment elevation were excluded from the experiment. Later, 24 adult female Wistar rats showing normal ECG were included in the study and divided into four groups each group containing six animals (*n* = 6). The experimental animals were grouped and treated as follows:

  Group I (normal healthy control): 2% dimethyl sulfoxide (DMSO) in double-distilled water; 1 ml/kg/day orally for 7 days + 0.9% NaCl, 1 ml/kg (single dose); i.p. on the 7^th^ day
  Group II (DOX control): DOX 25 mg/kg (single dose); i.p. on the 7^th^ day
  Group III (test A; DOX + MG 150 mg/kg): MG 150 mg/kg/day orally for 7 days + DOX 25 mg/kg (single dose); i.p. on the 7^th^ day
  Group IV (Test B; DOX + MG 300 mg/kg): MG 300 mg/kg/day orally for 7 days + DOX 25 mg/kg (single dose); i.p. on the 7^th^ day
  On the 8th day (24 hours after the administration of doxorubicin), all the experimental animals were anaesthetized by intraperitoneal administration of both ketamine (60 mg/kg) and xylazine (10 mg/kg)

### 2.6. ECG Recording

Following anaesthesia, each rat was positioned on an animal operation table for ECG recording. Electrodes were tied on the palmer surface of clean-shaven limbs of rats. The front limbs and left hind limb were used for recording of ECG in standard leads, while the right hind limb was attached with a grounded electrode. A conductive ECG gel was applied with care over each electrode to prevent a gel bridge between them from being formed. ECG (BPL Cardiart 9108) was recorded using lead II. For each animal, ECG was recorded for one minute, and only average of data from 11 consecutive ECG signals was used in the analyses. ECG of each experimental animal was analysed both qualitatively and quantitatively and further verified by an interventional cardiologist. ECG was analysed quantitatively in terms of the PR interval, QT interval, QTc interval, and QRS-complex amplitude. The *P* wave and ST segment were analysed qualitatively.

### 2.7. Collection of Blood and Serum Preparation

Subsequent to ECG recording, 2 ml blood was collected from the retro-orbital venous plexus of each anaesthetized rat by using capillary tubes. Blood was stored in microcentrifuge tubes, and following clot formation, serum was obtained by centrifuging the whole blood at 3,000 rpm for 20 minutes at 4°C using a Remi C-24 refrigerated centrifuge. Serum was stored at −80°C for further biochemical investigations.

### 2.8. Collection of the Heart and Its Gross Examination

Anaesthetized animals were euthanized after the blood collection. Animals were placed in a recumbent supine position on the animal operation table. An incision was made on the ventral aspect of the thoracic wall just above the diaphragm by using a surgical scalpel, and the thoracic cavity was opened. The heart was collected from the mediastinum by dissecting it out from the major blood vessels. Gross examination of the heart was performed. The heart was then washed in regular saline, soaked on blotting paper to extract the blood, and then, set for histopathological analysis in 10% formalin.

### 2.9. Estimation of Lipid Profile, CK-MB, CK, LDH, and AST

Serum lipid profile, CK-MB, CK, LDH, and AST were estimated using a semiautoanalyzer (Star 21 Plus, Mumbai, India) as per the standard protocol given along with the respective commercially available kits.

### 2.10. Estimation of Malondialdehyde (MDA) and Reduced Glutathione (GSH)

Serum was analysed for both MDA and GSH as per the protocol given by Satyam et al. [34, 35]. The optical density was read at 540 nm and 412 nm for MDA and GSH, respectively, using an iMark microplate absorbance reader (Bio-Rad laboratories, United States). Serum MDA and GSH level were calculated based on their absorbance and both were expressed as mM/ml.

### 2.11. Histopathological Analysis

After 24 hours of fixation of heart tissues in 10% formalin, tissue samples were dehydrated subsequently in 50% ethanol for 48 hours, in 70% ethanol for 48 hours, in 90% ethanol for 24 hours, and in 100% ethanol for 24 hours. Thereafter, heart tissues were kept in xylene till the tissues become transparent. Later, tissues were embedded in paraffin wax to prepare the block by using embedding rings, and tissue blocks were kept at −18°C for 24 hours. Then, histological sections of 5 *μ*m thicknesses were taken by using a rotary microtome. The sectioned tissues were kept in water bath for fixing in lysine-coated slides. Thereafter, slides fixed with tissues were dried in a hot plate, and following that, staining was performed using Haematoxylin and Eosin (H & E). All the pathological findings were verified by a pathologist.

### 2.12. Statistical Analysis

Using the Statistical Package for the Social Sciences (SPSS version 16.0; SPSS), data gathered at the end of experiment were expressed as mean ± standard deviation and analysed by one-way analysis of variance (ANOVA) followed by post hoc Tukey's test. A level for *p* ≤ 0·05 was considered to be statistically significant (*p* ≤ 0.05).

## 3. Results

### 3.1. Effect on ECG

**Figure 1:**
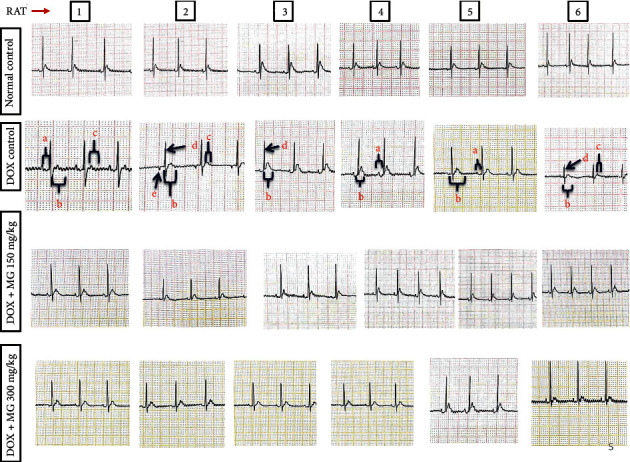
Qualitative analysis of ECG of all the experimental groups: a: increased PR interval, b: prolongation of the QT interval, c: nonspecific ST segment, d: reduced QRS complex amplitude, and e: inverted *P* wave.

Baseline ECG screening of some animals revealed depressed ST segment/absence of P wave/inverted P wave/nonspecific ST segment/ST segment elevation which were excluded from the experiment. Inverted P wave/increased PR interval/prolongation of QT and QTc interval/reduced QRS complex amplitude/nonspecific ST segment were observed among DOX control animals. However, these changes were not seen in normal control and test groups (Figure 1).

**Figure 2:**
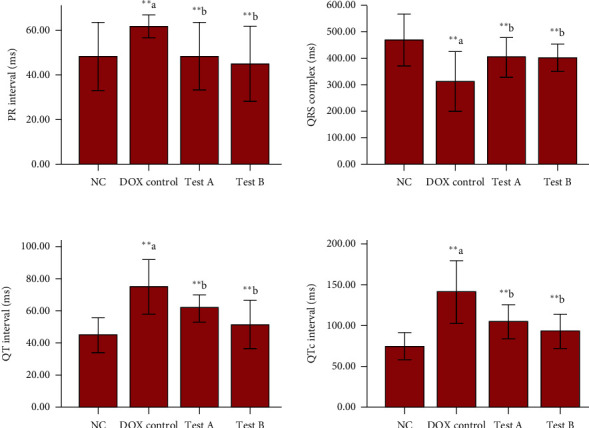
Quantitative analysis of ECG. Data were represented as mean ± SD, one-way ANOVA followed by Tukey's post hoc test ( ^*∗∗∗*^*p* ≤ 0.001,  ^*∗∗*^*p* ≤ 0.01, and  ^*∗*^*p* ≤ 0.05; ^a^compared to control and ^b^compared to DOX control), NC : normal control, test- A: methyl gallate 150 mg/kg, and test-B: methyl gallate 300 mg/kg.

There was a significant increase in the PR interval (*p* = 0.010), QT interval (*p* < 0.001), and QTc interval (*p* < 0.001) and decrease in QRS complex amplitude (*p* < 0.001) among DOX control animals in comparison with normal control animals. The PR interval (*p* = 0.010), QT interval (*p* = 0.010), and QTc interval (*p* = 0.006) were significantly decreased, and QRS complex amplitude (*p* = 0.006) was increased among the test-A-treated (DOX + MG 150 mg/kg) group compared to the DOX control group. A significant decrease in the PR interval (*p* = 0.002), QT interval (*p* < 0.001), and QTc interval (*p* < 0.001) and an increase in QRS complex amplitude (*p* = 0.010) in the test-B (DOX + MG 300 mg/kg) group in comparison with DOX control animals were also observed (Figure 2).

### 3.2. Effect on Serum CK-MB, CK, LDH, and AST Levels

**Figure 3:**
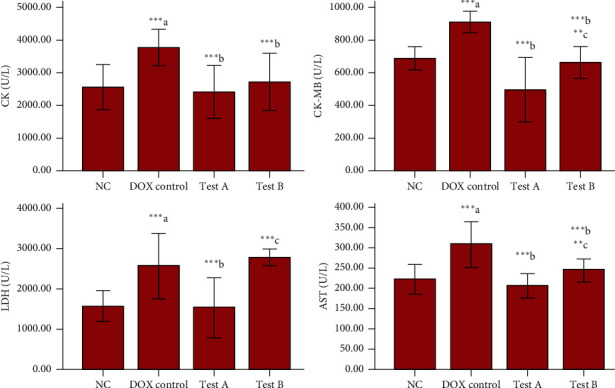
Effect of DOX and methyl gallate on cardiac injury biomarkers. Data were represented as mean ± SD, one-way ANOVA followed by Tukey's post hoc test ( ^*∗∗∗*^*p* ≤ 0.001,  ^*∗∗*^*p* ≤ 0.01, and  ^*∗*^*p* ≤ 0.05; ^a^compared to control, ^b^compared to DOX control, and ^c^compared to methyl gallate 150 mg/kg), NC : normal control, test- A: methyl gallate 150 mg/kg, and test-B: methyl gallate 300 mg/kg.

A significant increase was observed for CK-MB (*p* < 0.001), CK (*p* < 0.001), LDH (*p* < 0.001), and AST (*p* < 0.001) in the DOX control group in comparison with normal control. Both the doses of MG have significantly decreased CK-MB (*p* < 0.001), CK (*p* < 0.001), LDH (*p* < 0.001), and AST (*p* < 0.001) compared to the DOX control group. MG 150 mg/kg has significantly decreased CK-MB (*p* = 0.010), LDH (*p* < 0.001), and AST (*p* = 0.010) compared to the test group administered with MG 300 mg/kg (Figure 3).

### 3.3. Effect on Serum Lipid Profile

**Figure 4:**
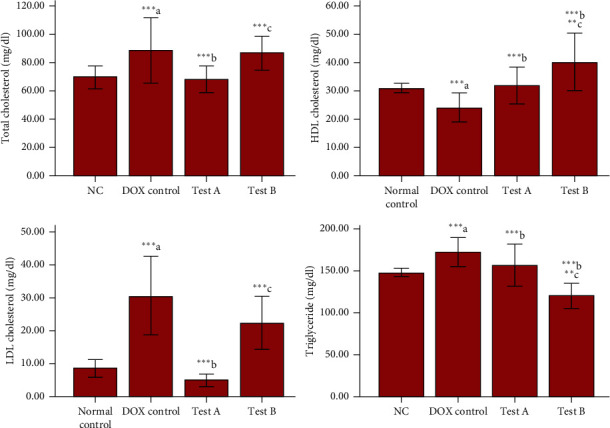
Effect of DOX and methyl gallate on lipid profile. Data were represented as mean ± SD, one-way ANOVA followed by Tukey's post hoc test ( ^*∗∗∗*^*p* ≤ 0.001,  ^*∗∗*^*p* ≤ 0.01, and  ^*∗*^*p* ≤ 0.05; ^a^compared to control, ^b^compared to DOX control, and ^c^compared to methyl gallate 150 mg/kg), NC: normal control, test- A: methyl gallate 150 mg/kg, and test-B: methyl gallate 300 mg/kg.

Triglyceride (*p* < 0.001), total cholesterol (*p* = 0.001), and low-density lipoprotein (LDL) cholesterol (*p* < 0.001) levels were significantly increased, and the high-density lipoprotein (HDL) cholesterol (*p* = 0.010) level was significantly decreased in the DOX control group compared to normal control. Animals treated with MG 150 mg/kg (test-A) had significantly low triglyceride (*p* = 0.025), total cholesterol (*p* < 0.001), and LDL cholesterol (*p* < 0.001) and significantly high HDL cholesterol (*p* = 0.004) compared to the DOX control group. Triglyceride (*p* < 0.001) was significantly reduced, and HDL cholesterol (*p* < 0.001) was significantly increased in MG 300 mg/kg-treated (test-B) rats in comparison with the DOX control group. MG 300 mg/kg has significantly reduced triglyceride (*p* < 0.001) and increased HDL cholesterol (*p* = 0.002) compared to the MG 150 mg/kg-treated group, whereas a significant decrease was found for total cholesterol (*p* = 0.001) and LDL cholesterol (*p* = 0.002) in MG 150 mg/kg-treated rats in comparison with test group MG 300 mg/kg (Figure 4).

### 3.4. Effect on Serum GSH and MDA Levels

**Figure 5:**
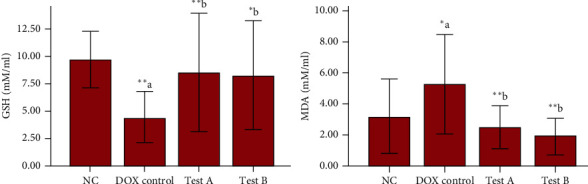
Effect of DOX and methyl gallate on oxidative stress markers. Data were represented as mean ± SD, one-way ANOVA followed by Tukey's post hoc test ( ^*∗∗∗*^*p* ≤ 0.001,  ^*∗∗*^*p* ≤ 0.01, and  ^*∗*^*p* ≤ 0.05; ^a^compared to control and ^b^compared to DOX control), NC: normal control, test-A: methyl gallate 150 mg/kg, and test-B: methyl gallate 300 mg/kg.

In the DOX control group, there was a significant decrease in GSH (*p* = 0.001) whereas MDA (*p* = 0.018) was significantly increased compared to normal control animals. MG 150 mg/kg (Test-A) has significantly increased GSH (*p* = 0.010) and decreased MDA (*p* = 0.002) levels in comparison with the rats treated with MG 300 mg/kg (Test-B). Similarly, there was a significant increase in GSH (*p* = 0.019) and decrease in MDA (*p* < 0.001) among the rats treated with MG 300 mg/kg compared to the DOX control group (Figure 5).

### 3.5. Gross Examination of Isolated Hearts

**Figure 6:**
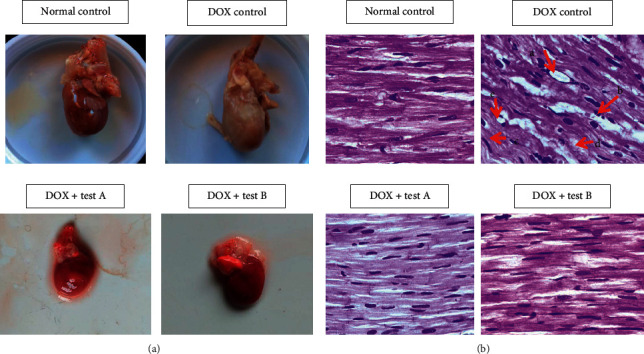
(a) Gross examination of the heart, (b) longitudinal section of cardiac tissue stained with H & E under 400X, (a) intermuscular edema, (b) infiltration with inflammatory cells, (c) myofibrillar loss, (d) cardiomyocytes degeneration, and (e) vacuolization.

Myocardial infarction (MI) as pale/yellow with hyperemic or hemorrhagic borders/white-grey (scar) was significantly noted among isolated hearts of DOX control rats, whereas these were absent in normal control and test groups (Figure 6(a)).

### 3.6. Histopathological Examination of Cardiac Tissue

DOX-induced cardiomyopathy was examined by using H & E stain under a light microscope. The normal control group had shown normal cardiomyocytes architecture. In the DOX-treated control group, cardiomyocytes had presented intermuscular edema, myofibrillar loss, infiltration with inflammatory cells, vacuolization, and cardiomyocytes degeneration. All these pathological changes were mitigated among the test-A and test-B group, and the cardiomyocytes architecture almost looked like the normal control group (Figure 6(b)).

## 4. Discussion

The present study demonstrates the cardioprotective potential of MG in female Wistar rats against DOX-induced cardiotoxicity. Acute cardiotoxicity caused by DOX is observed during and within 2-3 days of its single-dose administration [36–38]. Studies have reported that the incidence of acute cardiotoxicity is approximately 11% [11, 39]. Mechanisms of the therapeutic effects of doxorubicin on tumour cells vary from those of its mechanisms of cardiotoxicity. Although comprehensive cardiotoxicity investigations caused by doxorubicin have been underway for decades, the exact mechanism has not been thoroughly elucidated yet.

Most of the studies emphasize the increase in free-radical-induced cardiac oxidative stress and ROS-caused damage [18–20, 37]. DOX has great affinity towards negatively charged phospholipid cardiolipin, which is abundant in the inner mitochondrial membrane and accumulates it within the mitochondria of cardiomyocytes [17]. Singal et al. reported that DOX reduces endogenous antioxidants and undergoes increase in lipid peroxidation that alters the cardiac function and with other toxicities [22]. DOX induces excessive formation of ROS in the mitochondria, which leads to oxidative damage to biological macromolecules, including lipids, proteins, and DNA, and disturbs cardiac cell membrane structure and functions [21].

The present study has demonstrated acute cardiotoxicity with the single dose of DOX administration to the experimental animals. We observed that DOX has significantly altered ECG waves in the form of an inverted p wave/increased PR interval/prolonged QT and QTc interval/reduced QRS complex amplitude/nonspecific ST segment. An altered membrane function due to DOX-induced lipid peroxidation might be responsible for most of the ECG changes. MG at both the doses (150 mg/kg and 300 mg/kg) has significantly mitigated these acute changes in ECG.

The absence or altered form of the P wave occurs in various cardiac arrhythmias, of which atrial fibrillation is the most common. [40–42]. Milliez et al. had reported that *P*-wave prolongation in Wistar rats might be associated with high susceptibility with supraventricular arrhythmias following myocardial infarction [43]. The PR interval reflects the distribution of depolarization from an atria to the heart ventricles [44, 45]. An analysis of the length of the PR interval is crucial in the diagnosis of atrioventricular blocks. The PR interval was reported to increase with DOX [46, 47].

Complex narrowing of QRS is related to supraventricular arrhythmias. Large QRS complex represents ventricular rhythms, as well as disruptions in intraventricular conduction can be seen in cardiac insufficiency, myocardial ischemia, and right and left bundle branch blocks. Wide QRS complexes were observed in DOX-treated rats [48]. Studies suggested that multiple myocardial infarctions may lead to decreased amplitude of QRS complexes because of cancellations and diminished electromotive force generation [49–51].

The ST segment reflects the time of depolarization of the ventricles and is defined as the time between the end of the QRS complex and the commencement of the *T* wave. Rat studies have shown major changes in the ST segment following myocardial infarction [52] and in myocardial ischemia [53], but specific criteria for substantial ST segment variations were not well known. DOX has been reported to produce nonspecific ST changes [50, 51]. Detection of the ST segment in rat ECG was stated to be difficult, as the *T* wave always increases in continuity with the S wave [54, 55].

The QT interval represents the time of the ventricular cardiomyocyte depolarization and repolarization. The pathological duration of this parameter indicates disturbances in the heart's electrical activity caused by an intrinsic heart disease or exogenous compound toxic effects. The prolonged QT interval is considered a useful indicator of the drug-induced cardiotoxicity [56, 57]. Many studies have revealed QT prolongation produced by DOX [46, 47, 58, 59]. It is well established that QT interval lengths depend on the heart rate (HR). A rise in the HR usually shortens QT as the ratio of systole and diastole lengths increases. Hence, a corrected QT interval (QTc), which takes into account changes in HR, is often used as a more objective parameter of ventricular depolarization and repolarization [60, 61].

Raised levels of CK, CK-MB, LDH, and AST in serum are considered as indicators of myocardial injury [11]. Dyslipidaemia refers to heart complications such as atherosclerosis, which can cause toxic events in cardiac tissues. Single-dose administration of DOX has been reported to cause dyslipidaemia in rats [62]. In our study, DOX has significantly elevated CK, CK-MB, LDH, and AST and produced dyslipidaemia. This confirms the cardiotoxicity induced by DOX in rats that is supported by gross examination of isolated hearts following histopathological changes observed in heart tissues of experimental animals. DOX induced these biochemical, histopathological changes that were brought back to normal by both the doses (150 mg/kg and 300 mg/kg) of MG. The significant increase in serum CK, CK-MB, LDH, and AST after DOX administration correlates with the previous reports which suggest that DOX-induced oxidative stress can lead to lipid peroxidation that is accompanied by the release of these enzymes into serum [38, 39, 63–66].

We also found that DOX administration lead to increase in MDA and decrease in GSH levels. These results were in agreement with the previous studies [64, 67, 68]. El-Sayed et al. stated that DOX induces oxidative damage to heart tissue that results in lipid peroxidation with the concomitant production of MDA and reduction of GSH content [38]. MG at both the doses (150 mg/kg and 300 mg/kg) has reversed DOX-induced alterations in MDA and GSH levels.

Studies suggest that pretreatment with gallic acid reduces the detrimental oxidative effects of myocardial infarction because of its antioxidant potential either by increasing the activity of antioxidant enzymes such as superoxide dismutase and catalase and/or by increasing the level of nonenzymatic antioxidant markers such as MDA and GSH [69–72]. All of these activities can inhibit the detrimental effects of free radicals on the integrity and function of myocyte membranes, and hence, the concentration of serum cardiac biomarkers, including CK, CK-MB, LDH, and AST, decreases after infarction in addition to attenuation of dyslipidaemia [71, 73–75]. Methyl gallate is the methyl ester of gallic acid, and it has been reported to decrease MDA and increase GSH in oxidative stress animal models [76, 77].

Elevated ROS level reduces the expression of nuclear factor erythroid 2-related factor (Nrf2), which increases the susceptibility of cells to oxidative stress and apoptosis [78]. Heme oxygenase-1 (HO-1) is one of the antioxidant response elements- (AREs-) regulated phase II detoxifying enzymes regulated by Nrf2, which catalyzes heme degradation to biliverdin, carbon oxide, and iron [79, 80]. In particular, HO-1 has most of the abundant AREs in promotion of Nrf2-regulated genes and has been stated to be very significant in preventing oxidative stress-induced disease [81, 82].

DOX has been reported to decrease Nrf2/HO-1 expression to induce oxidative stress-mediated injury [83–85]. Studies have shown that Nrf2 activation could counteract DOX-induced cardiotoxicity via suppression of DOX-induced oxidative stress and impairment of autophagy [86–88]. Enhancing the critical biological functions of Nrf2 should be a safe and effective strategy to counter DOX-induced cardiotoxicity. Many studies have reported that MG modulates the Nrf2/HO-1 pathway and, thereby, protects from DOX-induced oxidative injury [31, 89, 90]. In this study, the inhibitory effect of MG on oxidative stress was demonstrated by a decrease in MDA and an increase in GSH levels, which indicates its anticipated cardioprotective mechanism. The protection against cardiac injury by MG through a possible membrane-stabilizing effect is supported by normalization of serum CK, CK-MB, LDH, and AST activities. A wide array of nutraceuticals such as quercetin, rutin, curcumin, epigallocatechin, genistein, apigenin, and resveratrol have been investigated as cardioprotectant. But, most of these compounds have variable bioavailability and wide drug interactions, and they decrease the anticancer effect of doxorubicin. This limits their use as cardioprotectant against doxorubicin against cardiotoxicity. We are conducting further studies to distinguish methyl gallate from available existing cardioprotectant nutraceuticals and abovementioned issues pertaining to them.

## 5. Conclusions

The present study revealed that MG exerts cardioprotection against DOX-induced cardiotoxicity in female Wistar rats by suppressing oxidative stress. Our study opens the perspective to clinical studies for consideration of MG as a potential chemoprotectant nutraceutical in the combination chemotherapy with DOX to limit its cardiotoxicity.

## Data Availability

All data arising from this study are included within the article.
